# Modulation of TonEBP activity by SUMO modification in response to hypertonicity

**DOI:** 10.3389/fphys.2014.00200

**Published:** 2014-06-19

**Authors:** Jeong-Ah Kim, Mi Jin Kwon, Whaseon Lee-Kwon, Soo Youn Choi, Satoru Sanada, Hyug Moo Kwon

**Affiliations:** ^1^Department of Biological Sciences, Ulsan National Institute of Science and TechnologyUlsan, South Korea; ^2^Department of Medicine, University of MarylandBaltimore, MD, USA

**Keywords:** TonEBP, renal medulla, transactivation

## Abstract

TonEBP is a DNA binding transcriptional enhancer that enables cellular adaptation to hypertonic stress by promoting expression of specific genes. TonEBP expression is very high in the renal medulla because local hypertonicity stimulates its expression. Given the high level of expression, it is not well understood how TonEBP activity is modulated. Here we report that TonEBP is post-translationally modified by SUMO, i.e., sumoylated, in the renal medulla but not in other isotonic organs. The sumoylation is reproduced in cultured cells when switched to hypertonicity. Analyses of site-directed TonEBP mutants reveal that K556 and K603 are independently sumoylated in response to hypertonicity. DNA binding is required for the sumoylation. Functional analyses of non-sumoylated mutants and SUMO-conjugated constructs show that sumoylation inhibits TonEBP in a dose-dependent manner but independent of the site of SUMO conjugation. Sumoylation inhibits transactivation without affecting nuclear translocation or DNA binding. These data suggest that sumoylation modulates the activity of TonEBP in the hypertonic renal medulla to prevent excessive action of TonEBP.

## Introduction

TonEBP (tonicity-responsive enhancer binding protein), also known as NFAT5 (nuclear factor of T cell 5), was originally identified as the key transcription factor that enables the cellular survival under hypertonic conditions (Miyakawa et al., 1999). TonEBP is a DNA binding protein that stimulates the transcription of a variety of genes involved in the survival under hypertonic conditions (Kwon et al., 2009). While some of these genes encode proteins involved in organic osmolyte accumulation, how other genes promote cellular adaptation to hypertonicity is poorly understood (Lee et al., 2011). Animals deficient in TonEBP activity display severe renal atrophy which is preceded by massive cell death in the renal medulla as cells fail to adapt to the local hypertonicity (Lopez-Rodriguez et al., 2004).

TonEBP is activated by hypertonicity in multiple pathways—nuclear translocation, enhanced transactivation, and increased abundance. Tonicity-sensitive nuclear localization signal in the TonEBP molecule is described for the hypertonicity-induced nuclear translocation (Kwon et al., 2008). Although domains of TonEBP involved in the hypertonicity stimulation are defined (Lee et al., 2003), it remains unclear how tonicity regulates these domains. Increased stability or half-life of TonEBP mRNA has been described to explain the enhanced expression of TonEBP in hypertonicity (Cai et al., 2005).

SUMO (small ubiquitin-like modifier) is structurally related to ubiquitin. SUMO modification or sumoylation is an essential post-translational modification that regulates protein functions including transcription, signal transduction, and DNA repair (Geiss-Friedlander and Melchior, 2007). In mammals, SUMO2 and SUMO3 are 97% identical to each other and about 50% identical to SUMO1 (Saitoh and Hinchey, 2000).

In the kidney medulla, expression of TonEBP stays very high due to local hypertonicity. Here we found evidence that the TonEBP activity was modulated by sumoylation in the renal medulla. TonEBP is sumoylated on two lysine residues in a manner dependent on ambient hypertonicity. Sumoylation inhibits the transactivation of TonEBP in a dose-dependent manner.

## Materials and methods

### Animal studies

Male Sprague-Dawley rats weight 180 g were purchased from Harland Sprague Dawley Inc., Indianapolis, IN, USA. All the procedures had been approved by the Institutional Animal Care and Use Committee of the University of Maryland. The animals were allowed to free access to water and food before they were euthanized for collection of kidneys and urine samples.

### Cell culture and transfection

HEK293 cells were maintained in Dulbecco's Minimum Essential Medium (Invitrogen, Carlsbad, CA) supplemented with antibiotics and 10% fetal bovine serum (Invitrogen). Cells were transfected using Lipofectamine2000 (Invitrogen). 0.25 μg of plasmid DNA in 250 μl of Opti-MEM (Invitrogen) was mixed with 5 μl of Lipofectamine2000 dissolved in 250 μl of Opti-MEM and incubated at room temperature for 20 min. The mixture was added to a 1.5 ml of trypsinized cell suspension containing 3.5 million cells in antibiotics-free culture medium, and seeded in a well of a 6-well cluster. After a day, the cells were cultured in isotonic or hyperosmotic medium made by addition of NaCl, sorbitol, or urea.

### DNA constructs

All the expression constructs were generated using standard cloning procedures and verified by restriction mapping and sequencing. A cDNA encoding the c-form of human TonEBP mRNA (Maouyo et al., 2002) was cloned into a mammalian expression vector pCMV-Tag2 to produce FLAG-tagged TonEBP. Site-directed mutants were made using the QuickChange XL site-directed mutagenesis kit (Stratagene, La Jolla, CA). SUMO fused TonEBP constructs were made by insertion of PCR amplified SUMO fragments (without the C-terminal di-glycine residues) between FLAG and TonEBP. Expression plasmids for Ubc9, Xpress-tagged SUMO isoforms, PIAS1, PIASxα were described previously (Choi et al., 2006; Wei et al., 2006). Expression vectors for mPIASy and mPIAS3 (Sramko et al., 2006) were kindly provided by Juraj Bies (NIH, Bethesda).

### Immunoblot and immunoprecipitation

For immunoblot detection of sumoylated TonEBP, prevention of proteolytic removal of SUMO was critical. Fresh rat tissues including brain, kidney, and lung were immediately homogenized for 30 s at full speed using Polytron (Brinkmann, Westbury, NY) in 30 volumes of hot (90–95°) lysis buffer (1.0% SDS, 1.0 mM sodium orthovanadate, and 10 mM Tris-Cl, pH 7.4). An aliquot was saved for protein assay using the BCA kit (Pierce, Rockford, IL). Concentrated Laemmli buffer was mixed and boiled for 5 min. As for HEK293 cells, washed and pelleted cells were homogenized in the hot lysis buffer by passing through a 24-G needle several times. TonEBP (Miyakawa et al., 1999) and FLAG antibody (Sigma, St. Louis, MO) were used for immunoblot analysis {1:2000}. For immunoprecipitation of TonEBP conjugated to the Xpress-tagged SUMO's, transfected HEK293 cells were homogenized in the hot lysis buffer and diluted 1:10 in IP buffer [150 mM NaCl, 1% Triton X-100, protease inhibitor cocktail (Roche, Indianapolis, IN), 30 mM Tris-Cl, pH 7.5]. The diluted homogenate was incubated overnight with Xpress antibody (Invitrogen) at 4° and the antigen-antibody complex was recovered using Protein G/A-agarose (Upstate, Billerica, MA).

### Luciferase assay

To assay the TonE-driven transcription, 0.8 × 10^6^ cells in suspension were transfected with 50 ng of TonE-driven *Photinus* luciferase expression construct (Colla et al., 2006) along with 100 ng of various combinations of empty vector plus TonEBP expression vectors in a well of 24-well cluster. After 20 h, the cells were switched to hypertonic medium or isotonic medium and cultured for an additional 4 h. Activity of luciferase from cell lysates was measured using a commercial kit, the Luciferase® Reporter Assay system (Promega, Madisone, WI). To analyze the transactivation of TonEBP, suspended cells were transfected with 200 ng of Gal4 upstream activation sequence-driven *photinus* luciferase expression construct (pFR-Luc), 50 ng of expression vector for GAL4-DBD (Gal4 DNA binding domain) fused to wild type or mutant TonEBP. The cells were treated and expression of luciferase was analyzed as above.

### RNase protection assay (RPA)

RNA was extracted from HEK293 cells using TriZol (Invitrogen). RPA probes were synthesized using T7 or SP6 RNA polymerase from the following cDNA's cloned in pCRII-TOPO (Invitrogen): human SMIT (nucleotides 1618–1967 of NM_006933) and human AR (corresponding to nucleotides 608–992 of NM_020299). The plasmid for synthesis of human β-actin probe was purchased from Ambion (Austin, TX). A commercial kit (Ambion) was used for RPA. Radioactivity of protected bands was visualized and quantified using a phosphorImager (BioRad, Hercules, CA). In each sample, the radioactivity of the SMIT or AR mRNA band was corrected for RNA loading by dividing with that of the β-actin mRNA band.

### Electrophoretic mobility shift assay (EMSA)

Cell extracts were prepared from HEK293 cells transfected with various TonEBP mutants using IP buffer (see above). Double-stranded DNA containing “hTonE” sequence (Miyakawa et al., 1999) was end-labeled using γ-[^32^P]-ATP. The cell extract (5 μg protein per reaction) was incubated for 10 min with 1 μg of poly(dA·dT) in 20 μl containing 20 mM HEPES (pH 7.9), 50 mM KCl, 5 mM MgCl_2_, 1 mM dithiothreitol, and 5% (vol/vol) glycerol. Where indicated 100 nM unlabeled hTonE was added. After addition of 1 nM [^32^P]-hTonE, the reaction was incubated for 20 min at room temperature. The mixture was electrophoresed for 2.5 h on a 4% polyacrylamide gel in 45 mM Tris, 45 mM boric acid, and 1 mM EDTA at 150 V. Radioactivity of the TonEBP bands were visualized and quantified using PhosphorImager.

### Statistical analysis

Where indicated data are expressed in Mean and s.e.m. with the number of independent experiments (n). *T*-test was performed using Microsoft Excel software.

## Results

### TonEBP is di-sumoylated in response to hypertonicity

**Figure 1:**
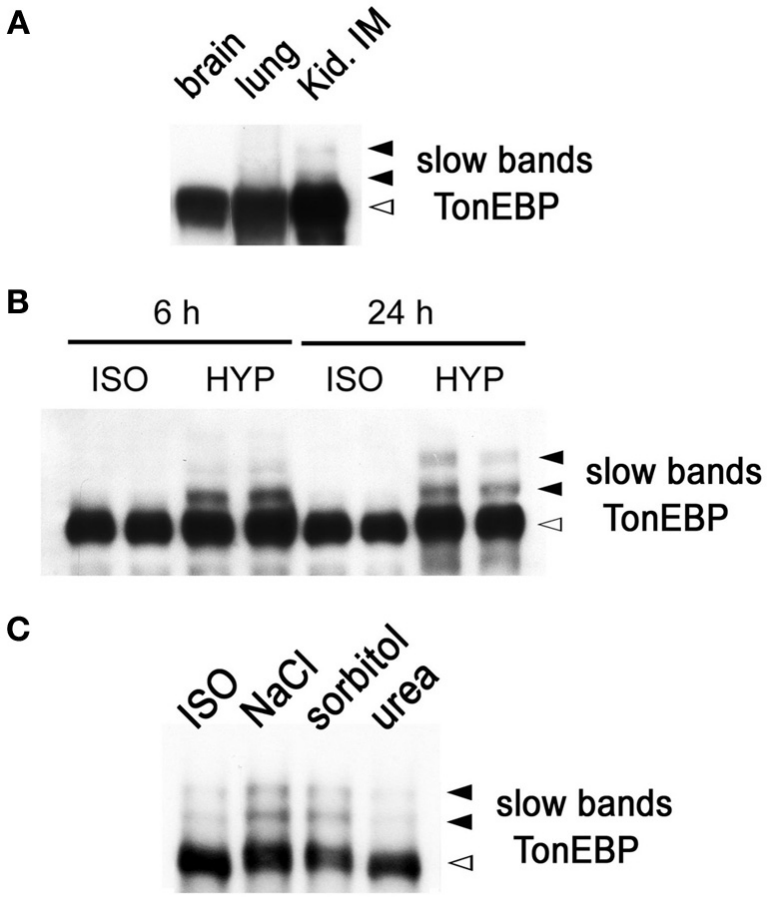
**Slow TonEBP bands in the kidney and HEK293 cells cultured in hypertonicity. (A)** A representative TonEBP immunoblot of brain, lung, and kidney inner medulla (Kid. IM) obtained from three euhydrated rats. Urine osmolality was 1730 ± 60 mosmol/kg. **(B)** TonEBP immunoblot of HEK293 cells cultured in isotonic (ISO) or hypertonic medium (HYP) for 6 or 24 h. **(C)** TonEBP immunoblot of HEK293 cells cultured in hyperosmotic medium containing additional 100 mM NaCl (NaCl), 200 mM sorbitol (sorbitol), and 200 mM urea (urea) for 6 h. TonEBP and slow bands are indicated on the right. The slow bands are 16 and 32 kDa larger than the TonEBP band.

While performing immunoblot analysis of TonEBP in animal tissues, we found that TonEBP was highly susceptible to proteolysis. To minimize the proteolysis, we have adopted the use of strong (1%) SDS in combination with immediate boiling for sample preparation as described in Materials and Methods. The new procedure yielded increased and reliable immunoblot signals for TonEBP. In addition, it came to our attention that two slow bands emerged in addition to the normal TonEBP band when samples from the outer (not shown) and inner medullae (Figure 1A) of kidneys were used. The slow bands were not seen in other tissues such as brain and lung (Figure 1A). Because the renal medulla is hyperosmotic, we tested whether hyperosmolality induced the slow TonEBP band. When HEK293 cells were cultured in hyperosmotic medium made by addition of 100 mM NaCl, the slow bands appeared clearly after 4 h and increased slowly over the course of 24 h (Figure 1B). The slow bands were also observed in other cell lines such as MDCK, HepG2, MEF, and RAW264.7 cells (data not shown). While NaCl or sorbitol was effective, urea was not effective in inducing the slow bands (Figure 1C) demonstrating that hypertonicity (effective hyperosmolality that causes water efflux from the cell) rather than hyperosmolality *per se* was the signal for the induction of the slow bands.

**Figure 2:**
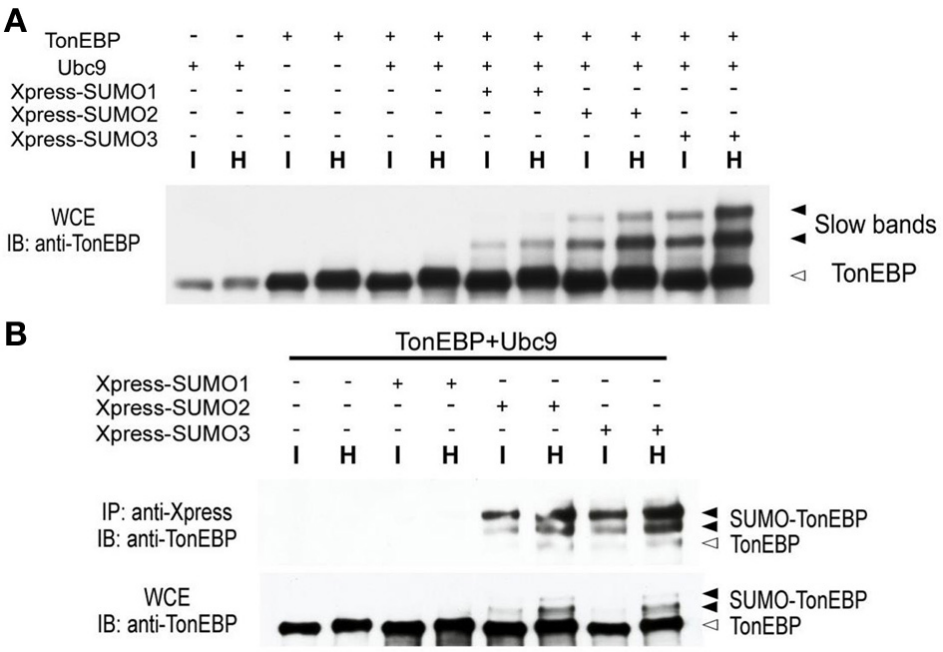
**TonEBP is sumoylated in response to hypertonicity. (A)** HEK293 cells were transfected with various combinations of TonEBP, Ubc9, and Xpress-tagged SUMO1, SUMO2, or SUMO3, as indicated. After 20 h, the cells were cultured for an additional 4 h in isotonic (I) or hypertonic medium (H). Whole cell extracts (WCE) were prepared and immunoblotted (IB) for TonEBP. Positions of TonEBP and slow TonEBP bands are indicated. **(B)** Cells were transfected as above and WCE were immunoprecipitated using anti-Xpression antibody. Immunoprecipitates (top) and WCE (bottom) were immunoblotted for TonEBP. Positions of TonEBP and sumoylated TonEBP (SUMO-TonEBP) bands are indicated at right.

Since the slow TonEBP bands were ~16 and ~32 kDa larger than TonEBP, we asked whether they were TonEBP molecules conjugated to SUMO, i.e., sumoylated TonEBP molecules. We found that overexpression of Ubc9, the only known E2 ligase for SUMO, promoted the formation of slow bands from endogenous TonEBP or transfected TonEBP (not shown; longer exposure the immunblot shown in Figure 2A revealed the slow bands in the first six lanes). Simultaneous overexpression of TonEBP, Ubc9, and Xpress-tagged SUMO isoforms resulted in a dramatic enhancement of the slow bands (Figure 2A). When the transfected cells were immunoprecipitated using antibodies against the Xpress tag, the slow bands were found in the pellets with TonEBP immuno-reactivity (Figure 2B) directly demonstrating that the slow bands were indeed sumoylated TonEBP molecules. The data in Figure 2 showed that SUMO3 and SUMO2 were far more efficient in sumoylation of TonEBP, and the sumoylation was stimulated by hypertonicity.

**Figure 3:**
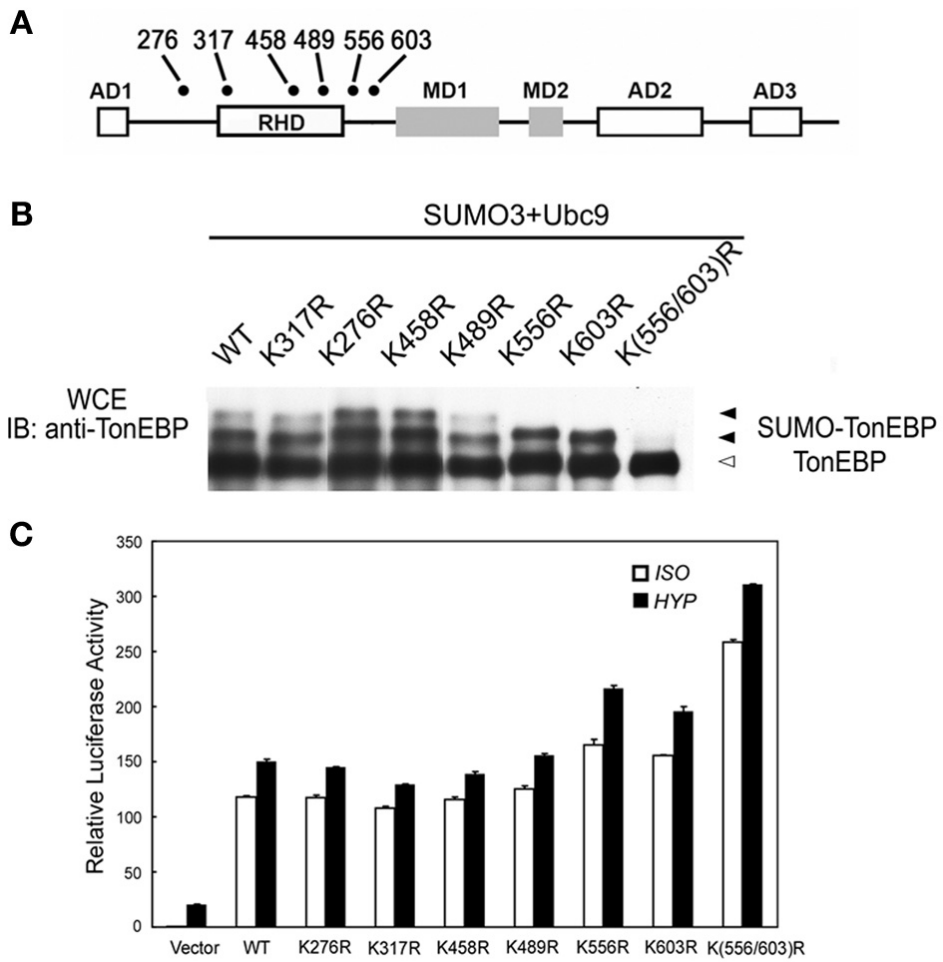
**TonEBP is sumoylated on lysines 556 and 603. (A)** Positions of lysine residues that fit the consensus sequence for sumoylation (ΨKxE: Ψ is a large hydrophobic residue; x is any residue) on a schematic primary structure of TonEBP. Rel homology domain (RHD), three activation domains (AD), and two modulation domains (MD) are shown. **(B)** HEK293 cells were transfected with wild type TonEBP (WT) or each of the TonEBP mutants shown along with SUMO3 and Ubc9. The cells were cultured for 4 h in hypertonic medium before immunoblot detection of TonEBP. Positions of TonEBP and sumoylated TonEBP (SUMO-TonEBP) are indicated. **(C)** HEK293 cells were co-transfected with a TonE-driven luciferase reporter plus empty expression plasmid (Vector) or expression plasmid containing WT or each of the TonEBP mutants as indicated. The cells were cultured for 4 h in isotonic (ISO) or hypertonic (HYP) medium before analysis of luciferase. Expression of luciferase activity is shown relative to isotonic vector control. K556R or K603R is significantly different from WT or K(556/603) both in ISO and HYP: *p* < 0.01. Mean + s.e.m., *n* = 6.

The majority of lysine residues conjugated to SUMO fit the consensus sequence: ΨKxE (Ψ is a large hydrophobic residue and x is any residue). There are six lysines in the human TonEBP molecule that fit the consensus as shown in Figure 3A. When individually mutated to arginine residues, only K556R and K603R mutants showed one slow bands while other mutants displayed two slow bands. When both residues were mutated (K556R/K603R), the remaining slow band disappeared demonstrating that TonEBP were di-sumoylated on K556 and K603. The activity of TonEBP increased in the single mutant and even higher in the double mutant (Figure 3C) suggesting that sumoylation inhibits TonEBP in a manner dependent on the stoichiometry of sumoylation (see more below). In addition, we concluded that sumoylations on the two lysine residues were independent of each other because mutation of one site did not affect sumoylation of the other. The sequence around K556 and K603 is conserved in all species from human to zebrafish suggesting that sumoylation of TonEBP is an important aspect of TonEBP regulation.

### Mechanism of hypertonicity-induced sumoylation

Recent studies have shown that phosphorylation is required for sumolyation in several transcription factors such as heat-shock factors, GATA-1, and myocyte enhancer factor two (Gregoire and Yang, 2005; Hietakangas et al., 2006). This is mediated by the phsophorylation-dependent sumoylation motif: ΨKxExxSP. The sequences around the two sumoylation sites of TonEBP appear to conform to the consensus: VK_556_KEISS_601_P and IK_603_SEDVT_608_P. Since TonEBP is phosphorylated in response to hypertonicity (Dahl et al., 2001; Lee et al., 2002), we examined the role of S600/S601 and T608. For this, we made phosphorylation-negative mutants—S600A/S601A and T608A—as well and phosphorylation-mimicking mutants S601D and T608E. However, sumoylation and transcriptional activity of these mutants were not affected by any of the mutations individually or in combination (data not shown). We conclude that phosphorylation of S600/S601 or T608 has no bearings on the sumoylation of TonEBP.

**Figure 4:**
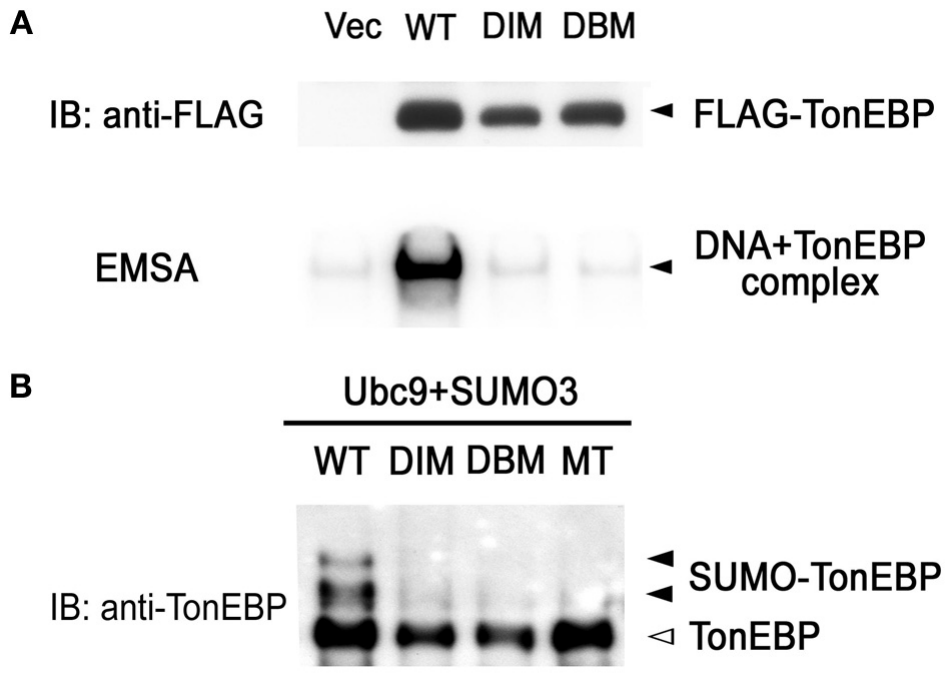
**DNA binding is required for sumoylation of TonEBP. (A)** HEK293 cells were transfected with empty vector (Vec) or one the following FLAG-tagged constructs: WT, wild type; DIM (dimer interface mutant), F464A/I466A; DBM (DNA binding mutant), T298A/E299A/R302A. Top: Cell lysates were immunoblotted for FLAG. Bottom: EMSA was performed using the cell lysates and 1 nM of [^32^P]-TonE. Positions of FLAG-TonEBP and DNA bound TonEBP are indicated. **(B)** HEK293 cells were transfected with Ubc9, SUMO3, and each of the constructs shown in **(A)** and MT (K556R/K603R mutant, see Figure 3). The cells were cultured for 4 h in hypertonic medium and immunoblotted for TonEBP.

Like NFκB, TonEBP forms a dimer and the dimerization is required for DNA binding (Lee et al., 2002; Stroud et al., 2002). We made site-directed mutants incapable of dimerization (DIM—dimer interface mutant: F464A/I466A) and incapable of DNA contact (DBM—DNA binding mutant: T298A/E299A/R302A). Coimmunoprecipitation analysis revealed that DIM was monomeric in solution while WT formed dimers (data not shown). As expected from the crystal structure of the Rel-homology domain of TonEBP (Stroud et al., 2002), DIM as well as DBM failed to bind DNA (Figure 4A). It should be pointed out that the sumoylation-negative mutant (MT in Figure 4B) bound DNA normally (see below). Interestingly, sumoylation was dramatically reduced in DIM and DBM as in MT (Figure 4B). These data provide evidence that DNA binding is required for the sumoylation of TonEBP. This raises the possibility that sumoylation of TonEBP takes place on the surface of chromatin when TonEBP is bound to DNA.

### Sumoylation inhibits TonEBP

**Figure 5:**
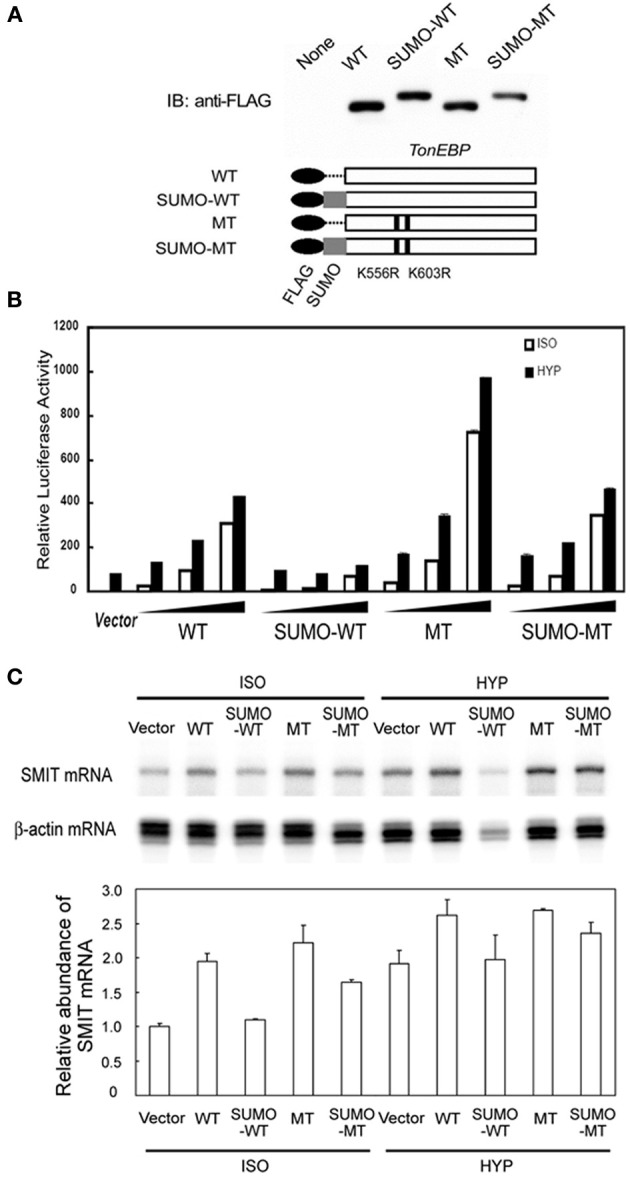
**Effects of sumoylation on the transcriptional activity of TonEBP. (A)** Schematics of FLAG-tagged TonEBP constructs are shown at bottom: wild type TonEBP (WT), SUMO3-fused TonEBP (SUMO-WT), K556R/K603R mutant TonEBP (MT), or SUMO3-fused MT (SUMO-MT). These constructs were transfected into HEK293 cells and immunoblotted for FLAG (top). **(B)** HEK293 cells were transfected with empty vector or increasing amounts of the constructs shown in A along with a TonE-driven luciferase reporter, and cultured for 4 h in isotonic (ISO) or hypertonic medium (HYP). Expression of luciferase activity is show as in Figure 3. **(C)** HEK293 cells were transfected with empty vector or each of the constructs shown in **(A)**. The cells were cultured for 6 h in isotonic (ISO) or hypertonic (HYP) medium. SMIT and β-actin mRNA's were measured by RPA. The radioactivity in each SMIT band was divided by that of corresponding β-actin band to correct for RNA loading. In ISO, WT was significantly different from SUMO-WT (*p* < 0.01) but not from SUMO-MT. Mean + s.e.m., *n* = 3.

The data shown in Figure 3C indicate that sumoylation leads to inhibition of TonEBP. To explore the functional consequence of the sumoylation further, we examined transcriptional activity of several TonEBP mutants along with WT (wild type TonEBP): SUMO-WT (SUMO3 was fused to WT to mimic sumoylated TonEBP), MT (K556R/K603R mutant that is incapable of sumoylation as shown in Figure 3), and SUMO-MT (MT fused to SUMO3) as schematically depicted in Figure 5A. WT stimulated the expression of TonE-driven reporter in a dose dependant manner in isotonic and hypertonic condition, as reported previously (Maouyo et al., 2002). SUMO-WT is practically inactive except for a small activity in isotonicity, while MT was two times more active than WT. Interestingly SUMO-MT displayed activity comparable to that of WT suggesting that WT was normally mono-sumoylated. Essentially the same effects were observed on the mRNA expression of TonEBP target genes, SMIT (Figure 5C) and AR (not shown), especially under isotonic conditions. Taken together, the data in Figure 5 demonstrate that sumoylation inhibits TonEBP in a manner dependent on the stoichiometry of sumoylation.

**Figure 6:**
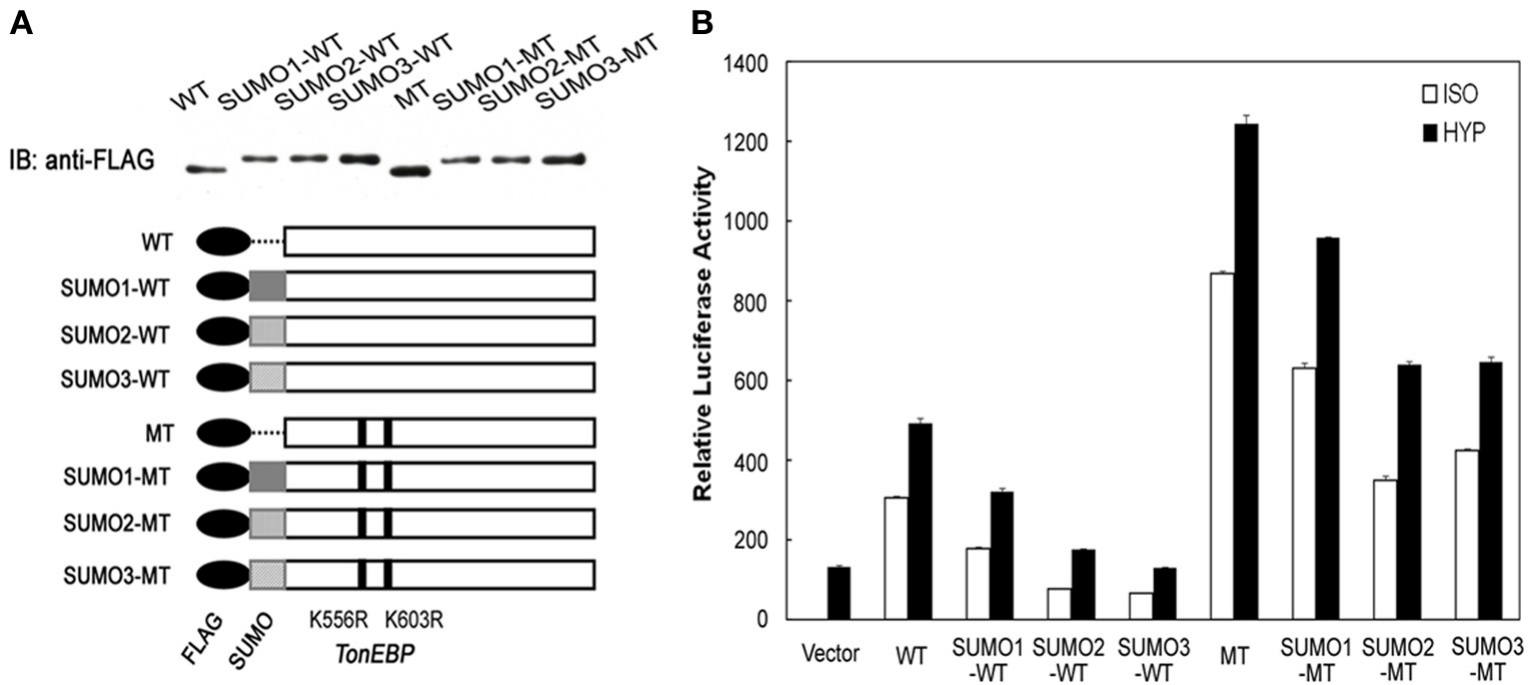
**SUMO isoform-dependent effects of sumoylation on the transcriptional activity of TonEBP. (A)** Schematics and expression of constructs are shown as in Figure 5A, except that SUMO1, SUMO2, and SUMO3 were used to make fusion proteins. **(B)** HEK293 cells were transfected and analyzed as in Figure 5B.

The data in Figure 2 showed that the efficiency of TonEBP sumoylation differed depending on the isoform of SUMO—SUMO1, SUMO2, and SUMO3—involved. Here we examined the different isoforms of SUMO on the efficiency of TonEBP inhibition. We made more SUMO fusion constructs using SUMO1 and SUMO2 in addition to SUMO3 as depicted in Figure 6A. While the conjugation of SUMO2 led to almost identical results to that of SUMO3, the conjugation of SUMO1 resulted in a smaller inhibition of TonEBP (Figure 6B). This is not surprising because SUMO2 and SUMO3 share 97% of amino acids while SUMO1 shares only 50% with SUMO2 or SUMO3 (Saitoh and Hinchey, 2000). Combined with the data in Figure 2, these results show that SUMO2 and SUMO3 are more effective in inhibiting TonEBP compared to SUMO1 as well as being more efficient in the sumoylation of TonEBP.

### Transactivation of TonEBP is inhibited by sumoylation

**Figure 7:**
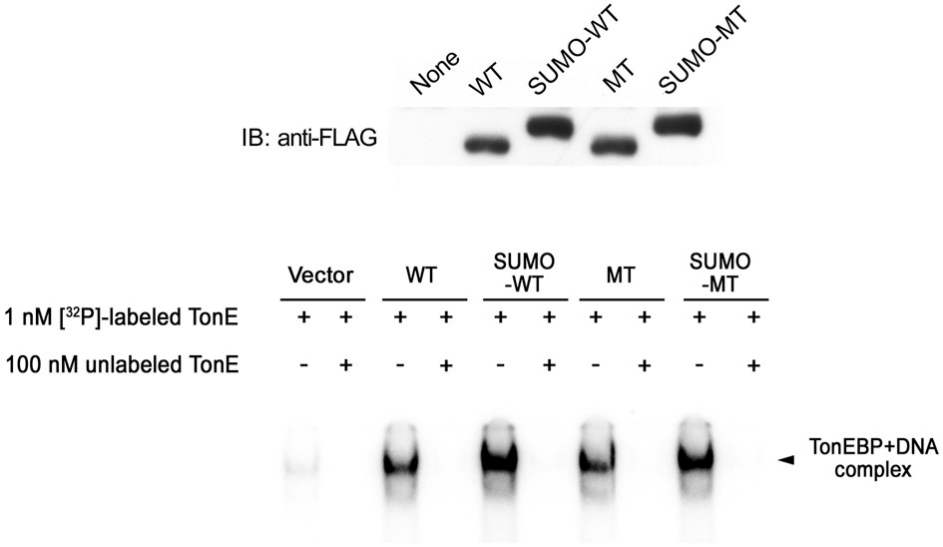
**Effects of sumoylation on DNA binding of TonEBP. Top:** HEK293 cells were transfected with the constructs shown in Figure 4A. Cell lysates were prepared and immunoblotted for FLAG. **Bottom**: EMSA was performed on the cell lysates using 1 nM [^32^P]-labeled DNA containing TonE in the absence or presence of 100 nM unlabeled DNA containing TonE as indicated (Bottom). DNA bound TonEBP is indicated.

We asked how sumoylation inhibited TonEBP using the non-sumoylated and sumoylation-mimicking TonEBP mutants discussed above. First, we examined the nuclear translocation of TonEBP in response to hypertonicity. We found that all the mutants displayed normal nuclear translocation (data not shown). Second, we found that the mutants bound TonE containing DNA like wild type TonEBP with comparable efficiency and affinity (Figure 7). Thus, sumoylation of TonEBP did not influence the nuclear translocation or DNA binding.

**Figure 8:**
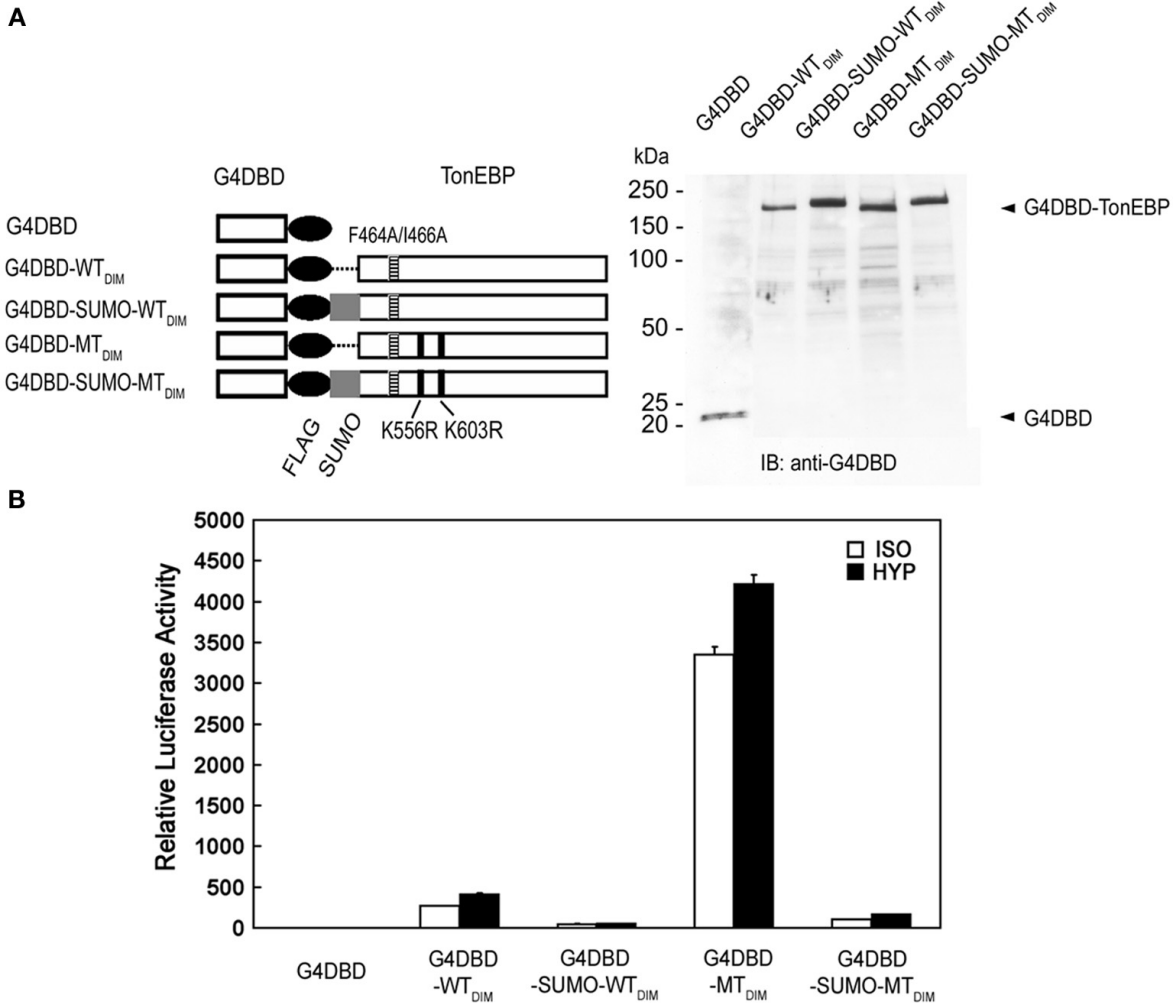
**Effects of sumoylation on the transactivation of TonEBP. (A)** Left: The DNA binding domain of Gal4 (G4DBD) was fused to the TonEBP constructs shown in Figure 5 except that F464A/I466A mutations (indicated by “_DIM_”) were introduced to prevent dimer formation. Right: The constructs were individually transfected into HEK293 cells and immunoblotted for G4DBD. **(B)** HEK293 cells were transfected with each of the constructs shown in **(A)** along with a Gal4-luciferase reporter. The cells were cultured for 4 h in isotonic (ISO) or hypertonic (HYP) medium before analysis of luciferase. Expression of luciferase activity is shown relative to isotonic vector control. Values in all the constructs are different from each other both in ISO and HYP (*p* < 0.01). Mean + s.e.m., *n* = 3.

Next, we examined the transactivation of TonEBP by employing fusion with the Gal4 DNA binding domain (G4DBD). To eliminate potential complications of dimer formation by TonEBP, dimer interface mutations (see Figure 4) were introduced into TonEBP as indicated in Figure 8A. We later found that introduction of these mutations did not affect the results, i.e., the results were the same without or with the dimer interface mutations (not shown). As shown in Figure 8B, wild type TonEBP displayed a strong transactivation, which was stimulated by hypertonicity as reported earlier (Lee et al., 2003). The transactivation was inhibited more than 80% by SUMO fusion (G4DBD-SUMO-WT_DIM_), but stimulated by several folds in the mutant incapable of being sumoylated (G4DBD-MT_DIM_). These data demonstrate that sumoylation is a powerful negative regulator for the transactivation of TonEBP.

## Discussion

In order to understand the slow TonEBP immunoreactive bands observed in the renal medulla, we investigated sumoylation of TonEBP. The slow bands are not observed in isotonic tissues like brain and lung. On the other hand, the slow bands are reproduced in cultured cells when the cells are subjected to hypertonicity. Because of the transient nature of sumoylation in general, we used overexpression of SUMO E2 ligase Ubc9 in combination with SUMO isoforms to demonstrate that TonEBP is di-sumolyated on lysine 556 and lysine 603. The sumoylation appears to take place in the nucleus because DNA binding is required. This is supported by the time course of sumoylation in response to hypertonicity which mirrors the previously reported time course of binding to the TonE sites (Miyakawa et al., 1998). Studies using site-directed TonEBP mutants incapable of sumoylation and SUMO-conjugated TonEBP constructs reveal that TonEBP is inhibited by sumoylation in a dose-dependent manner regardless of the position of SUMO conjugation on the TonEBP molecule. Sumoylation inhibits TonEBP by reducing its transactivation without affecting nuclear translocation or DNA binding. From these data we can envision the following scheme of events after a cell is exposed to hypertonicity. Initially the nuclear TonEBP abundance rises due to enhanced expression in combination with nuclear translocation leading to DNA binding and expression of TonEBP target genes. Later, the TonEBP molecules bound to DNA are sumoylated and their transactivation decreases. Such tempering of TonEBP activity might be important for homeostasis in the renal medulla where TonEBP expression is very high most of the time. It was reported previously that the high concentration of nitric oxide in the renal medulla also inhibits TonEBP by direct *S*-nitrosylation (Neuhofer et al., 2009). Thus, the high level of TonEBP expression in the renal medulla is counterbalanced by inhibitory post-translational modifications including sumoylation and *S*-nitrosylation.

In many other transcription factors whose transactivation is inhibited by sumoylation, the underlying mechanism involves recruitment of histone deacetylases (HDAC's) and interaction with co-repressors (Gill, 2005). Indeed, we found that TonEBP was co-immunoprecipitated with HDAC1 (data not shown). However, the interaction was intact in non-sumoylated and SUMO-fusion constructs suggesting that sumoylation did not lead to recruitment of HDAC1. In addition an inhibitor of HDAC1, trichostatin A, did not affect the transactivation of non-sumoylated and sumoylation-mimicking mutants (not shown). It appears that the inhibition of transactivation by sumoylation is mediated by factors other than deacetylation.

### Conflict of interest statement

The authors declare that the research was conducted in the absence of any commercial or financial relationships that could be construed as a potential conflict of interest.
